# Modern public health problems and solutions: An undergraduate capstone course to prepare the next generation of public health practitioners to enhance health equity

**DOI:** 10.3389/fpubh.2022.992835

**Published:** 2022-10-05

**Authors:** Albina N. Minlikeeva, Katharine A. Amato, Sarahmona M. Przybyla

**Affiliations:** ^1^Department of Epidemiology and Environmental Health, School of Public Health and Health Professions, University at Buffalo, Buffalo, NY, United States; ^2^Department of Community Health and Health Behavior, School of Public Health and Health Professions, University at Buffalo, Buffalo, NY, United States

**Keywords:** capstone course, undergraduate public health education, bachelor of science public health, health equity, public health workforce

## Abstract

With a growing emphasis on health equity in public health practice and research, ensuring a competent and skilled public health workforce is critical to advancing the public health mission of a healthier nation. The expansion of undergraduate public health programs provides a unique opportunity for more extensive training and education of the next generation of public health professionals and to center undergraduate public health education around the need to be competent in addressing health disparities to achieve health equity. Following national accreditation standards set by the Council on Education for Public Health (CEPH), undergraduate Bachelor of Public Health (BSPH) students at the University at Buffalo (UB) must complete a capstone course before graduation. This course focuses on integrating and synthesizing knowledge acquired from the BSPH core curriculum through analysis, explanation, and addressing public health problems *via* an interdisciplinary approach. We designed the most recent iterations of the capstone class based on the model that includes cross-cutting skills as defined by CEPH, evidence-based decision-making skills, established learning objectives of the course, and centering on health equity. This course also builds on the students' previously acquired knowledge with an ultimate goal to prepare the graduating seniors for the “real world” health equity-related public health activities. As a part of the coursework, students complete case studies, article reviews, and active learning group activities that target each component of the model. The final products of the course are a synthesis paper and oral presentation based on a public health problem as identified through surveillance data, analyzing causes of this problem, identifying critical stakeholders, creating an evidence-based solution to the problem, and explaining how health inequities may be addressed through the proposed solution. Centering the culminating course for BSPH undergraduate students on health equity will help ensure a competent and skilled workforce, informed by accreditation standards and prepared to lead our national public health goal of improved and equitable population health.

## Introduction

Recently there has been a considerable shift toward health equity being a central theme of public health-related activities. Achieving health equity and addressing health disparities to improve people's health are the main goals of Healthy People 2030 (1). Similarly, the most recent version of the Essential Public Health Services was centered around the theme of health equity (2). The practical implications of these initiatives are reflected in the work of the leading public health agency in the country, the Centers for Disease Control and Prevention, being oriented around health equity (3).

The expansion of undergraduate public health programs provides a unique opportunity for more extensive training and education of the next generation of public health practitioners. As such, schools and programs of public health have a prime opportunity to center undergraduate public health education around themes that reflect modern public health practice, which includes emphasizing the critical role of health equity. In the capstone course of the BSPH program, we structured the coursework around a model that prepares the graduating seniors to participate in the important task of achieving health equity by targeting the cross-cutting skills developed and required by the Council on Education for Public Health (CEPH) and using the coursework as a mode of exposing the students to these concepts (4).

### Background and course rationale

The Undergraduate Public Health Program in the School of Public Health and Health Professions at the University at Buffalo (UB) started in 2017 and has considerably expanded over the years (5). The curriculum of the Bachelor of Science in Public Health (BSPH) program is based on an interdisciplinary approach that integrates five foundational public health disciplines in each core course of the curriculum (5). The details of this program and, specifically, the structure of the interdisciplinary curriculum have been described elsewhere (5). Briefly, the program has seven required courses: PUB 101- Introduction to Public Health, PUB 102- Historical and Contemporary Public Health Problems, PUB 310-Health and Disease: Biological, Personal, and Environmental Influences, PUB 315- Asking and Answering Scientific Questions in Public Health, PUB 320- Models and Mechanisms for Understanding Public Health, PUB 325- Interventions to Address Public Health Problems, PUB 330- Public Health Systems and Policies, and five elective courses (two at the 200-level and three at the 300/400-level). In the final semester of study, all BSPH students also complete PUB 494- Modern Public Health Problems and Solutions. The core public health courses correspond to the Public Health Bachelor's Degree Foundational Domains and Foundational Competencies and incorporate the Public Health Bachelor's Degree Cross-Cutting Concepts and Experiences required by CEPH (4, 5).

The students also have to complete additional coursework that includes a Communication Literacy course and some courses from other disciplines (5, 6). The structure of the program is built on the integration of the five main public health disciplines across the core courses (5). The interdisciplinary nature of the BSPH program prepares the students to establish and develop the knowledge and skills required to understand and analyze complex public health problems and develop solutions to address these problems using a “real world” approach (5).

The capstone course, PUB 494: Modern Public Health Problems and Solutions, is a four-credit course that represents one of the pivotal components of the BSPH curriculum (5). The course satisfies the D11 CEPH requirement, according to which the students “have opportunities to integrate, synthesize and apply knowledge through cumulative and experiential activities”(4). More specifically, in this course, the students have to demonstrate their ability to integrate and synthesize knowledge obtained from the public health curriculum and use that knowledge to analyze, explain, and address various public health problems (5).

The first offering in this course occurred in Spring 2019 with 17 students enrolled in this course. Over time, as the program expanded, the number of students enrolled in this course also increased. In Spring 2022, there were 102 students registered for this course, with four sections. This course is also offered during the Fall and Summer terms.

## Pedagogical framework

The capstone course enrolls students with diverse backgrounds and different career plans. In Spring 2022, to gather more information on the career plans of the graduating seniors of the BSPH program, we conducted an anonymous, self-administered survey. Survey results (response rate=78.4%) demonstrated racial and ethnic diversity in the sample, with 60 %White, 16.2% Black or African American, and approximately 11% indicating their ethnicity as Hispanic (Table 1). These numbers are very similar to the results of the most recent “Public Health Workforce Interests and Needs Survey: PH WINS” that was conducted among state and local public health workers in 2017. Based on this survey, the PH workforce is 59% of White, 15% Black or African American, and 13% Hispanic or Latino (7).

**Table 1:** Characteristics of graduating seniors of the BSPH program taking the capstone course in the spring 2022, *N* = 80.

**Characteristics**	***N* (%)**
**Gender**	
Female	61 (76.2)
Male	17 (21.2)
Other	2 (2.5)
Age (mean), years	22
**Race**	
White	48 (60.0)
Black/African American	13 (16.2)
Asian	12 (15.0)
American Indian/Alaskan Native	1 (1.3)
Decline to answer/unknown	6 (7.5)
**Ethnicity**	
Hispanic	9 (11.3)
Non-Hispanic	69 (86.2)
Decline to answer	2 (2.5)
**Plans after graduation**	
Continuing education	44 (55)
Enter the workforce	18 (22.5)
To be determined	9 (11.3)
Taking a break	9 (11.3)

In our sample, when asked about plans after graduation, more than half (55%) of students were planning to continue their education, while 22.5% indicated that they plan to enter the workforce, while more than ten percent (11.3%) said that their plans are not definite, and another 11.3% indicated that they would be taking a break (Table 1). Out of those who intended to continue their education, the majority of students were planning on pursuing the MPH degree or the Public Health Certificate Program. Some students indicated their interest in pursuing a degree in a health-care-related field such as nursing, while others were also interested in exploring other master's level degrees. Such a variety of potential career paths is not surprising as it reflects the multidisciplinary nature of public health (8).

### Course model

**Figure 1:**
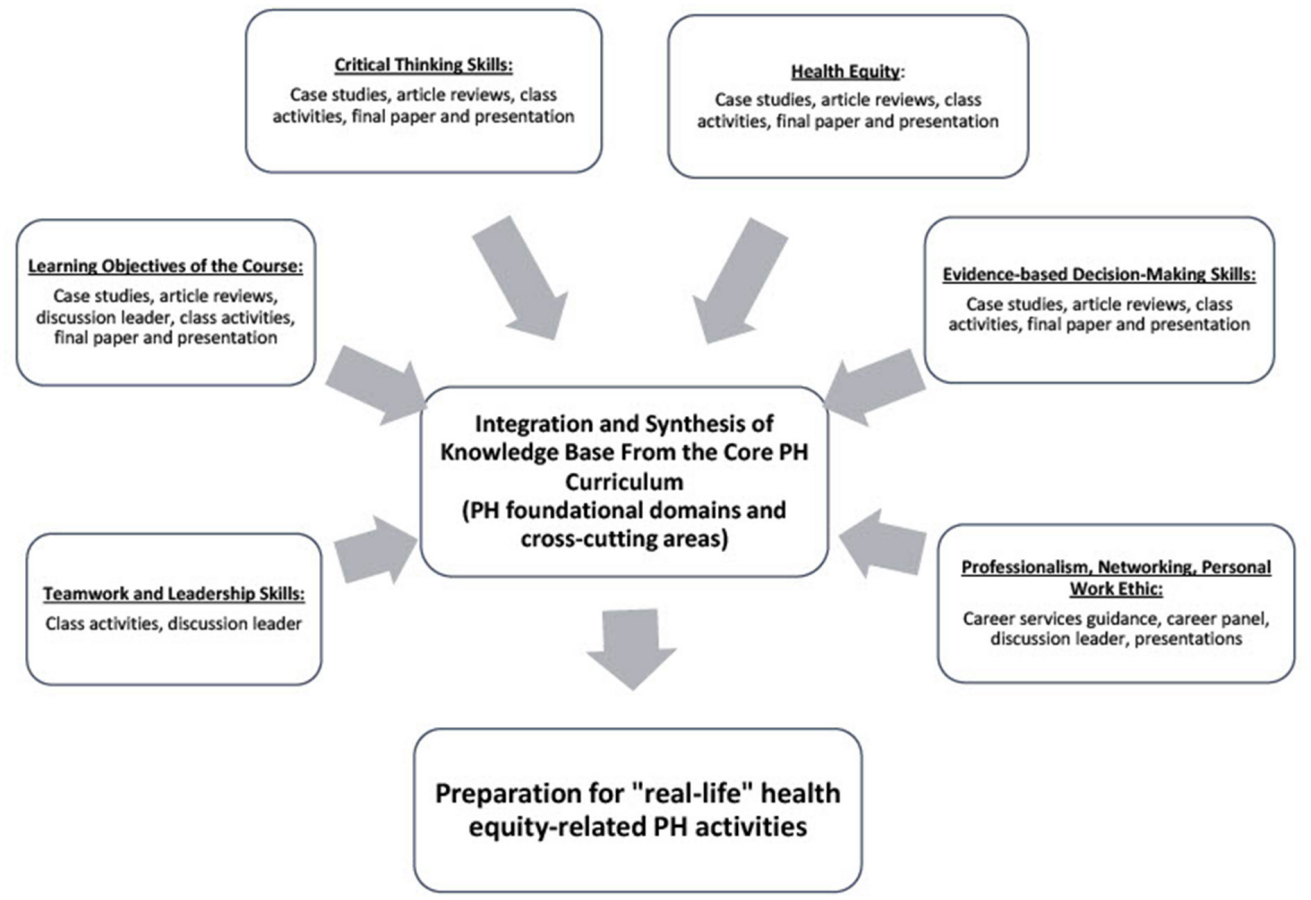
Model utilized in the modern public health problems and solutions class*. Adapted from Andreasen (9).

The coursework structure is reflected in the model depicted in Figure 1. This model originated from Randall Andreasen's work which described a Model for the Integration of Experiential Learning into Capstone Courses utilized in the College of Agriculture Capstone Courses (9). We adapted this model to be utilized in our course and modified it to reflect the focus of our class on the CEPH requirements described above and the overall public health focus on health equity (Figure 1). In addition, the model also incorporated the multidisciplinary structure of public health and accommodated skills needed for various career paths considered by the BSPH graduates. More specifically, the course targets several cross-cutting areas that are important for future professional success: professionalism, personal work ethic, networking, teamwork and leadership, and critical thinking (4). Another component targeted by this model is evidence-based decision-making skills. Emphasis on evidence-based practice and mastery of evidence-based decision-making skills is particularly important in this context, considering the leading role of scientific evidence in public health practice (10) and the emphasis of the use of evidence-based interventions and strategies to achieve health equity by Healthy People 2030 (1).

Another component integrated into the coursework is the course learning objectives (Figure 1) which include the following: (1) describing the connections between understanding of and solutions to public health problems, (2) explaining how evidence-based practice is implemented across the ten essential public health services (EPHS), (3) describing how the knowledge base of public health is used by “real world” public health practitioners, and (4) applying the knowledge base from the major core curriculum to comprehensively analyze, explain, and develop a plan to address a public health problem. The latter is directly related to the CEPH requirement for the capstone course in undergraduate public health programs.

Finally, another component that becomes a significant part of this model is its emphasis on health equity. While this concept is addressed in part by the second learning objective of the course as part of the ten EPHS, in this model, we decided to distinguish this concept from the learning objectives and separate it into a distinct component of this model.

To fulfill the CEPH requirements for the course, these components of the model are aligned around the integration and synthesis of knowledge gained in the major core public health curriculum. To accomplish this goal, each component of the model also includes the methods of evaluating students' achievements presented by case studies, article reviews, class activities, leading discussions, and final paper and presentation. The description of the “Professionalism, networking, and personal work ethic” component also includes career panel and career services guidance. These will be described in the next section of the paper.

To reflect the overall goal of public health initiatives and the need for the public health workforce to be knowledgeable in health equity, the building blocks of the model listed above are all connected to the final component titled “preparation for health equity-related public health activities” which emphasizes the special role of health equity topic in this course.

## Pedagogical format

### Professionalism, networking, and personal work ethic

The first part of the course aims to address the students' different needs and career-related aspirations because of the variety of different career and professional paths considered by the students. Specifically, to prepare the students for the next step after graduation and address the issue of preparation for future career requirements, the course starts with a series of career development workshops in which the career services representatives provide guidance on topics such as networking, job searching, interviewing, and job negotiation skills. During these talks, the importance of one's personal work ethic is also highlighted. The students are provided information on resume, CV, and cover letter writing as well as guidance on professional communication. The students are also linked to the UB Career Design Center, where they can utilize the services and tools available to them to prepare for the next steps after graduation beyond what has been discussed in the classroom.

Students also interact with a public health practitioner career panel, which consists of recent alumni of the BSPH program at UB, graduate students currently enrolled in various concentrations of the MPH degree, and public health professionals with MPH degrees. During the session, the panelists share their post-graduation and work-related or classroom experiences and answer questions regarding topics such as future career steps, the importance of networking, professionalism, and a strong work ethic.

### Learning objectives of the course, evidence-based decision-making skills, and health equity

In capstone, the coursework integrates the knowledge and experiences acquired by the students in the previous classes that target various public health foundational domains, foundational competencies, and cross-cutting concepts and experiences. The students start the class equipped with the foundational public health-related knowledge from other public health courses that include but are not limited to the topics related to quantitative and qualitative research methods, public health biology, behavioral models, social determinants of health, health policies and laws, planning and implementation of public health interventions, and searching for and evaluation of peer-reviewed literature related to public health content.

Throughout the semester, the students are exposed to multiple activities, case studies, and articles assigned by the instructors. To be aligned with the learning objectives of the course and centered on health equity, the class activities and assignments are structured around the revised 2020 version of the ten Essential Public Health Services (EPHS) (2). Each week, one of the ten EPHS and a corresponding function of public health becomes the main topic for course-related activities and assignments. Students complete case studies, article reviews, and active learning class activities related to this EPHS. When completing each of these assignments and activities, students are exposed to discussion and detailed analysis of various public health problems that focus on infectious and chronic diseases, health-related behaviors, and environmental issues and public health-related solutions that include interventions, policies, and programs. Particular attention is devoted to discussing the role of stakeholders involved in the problem and solution of this problem and the interaction of these stakeholders during the implementation phase of the solution. Such detailed focus on each EPHS and public health efforts to maintain these EPHS improves the understanding of the practical implications of EPHS and a corresponding public health function. It also increases awareness of the role of evidence-based practice in public health and strengthens evidence-based decision-making skills. For example, when working on one case study focused on chronic disease, the students were asked to explain how evidence was used in this case study to understand the problem, create the solution, guide the decision on what stakeholders to involve in the solution to the problem and evaluate the results of a specific intervention based on available evidence (11).

Besides being focused on the EPHS, one of the main themes that become a centerpiece of these assignments and discussions is health equity. For each assignment, students demonstrate their knowledge of health equity and explain how each of the discussed scenarios and each EPHS is linked to health equity. The students also explore the absence of health equity, how the EPHS can help address health inequities, and creating solutions aligned with the EPHS to address health inequities. Such a structure allows students to practice approaching different public health problems and their solutions through a health equity lens.

### Teamwork and leadership skills

Another aspect targeted by the coursework is the mastery of teamwork and leadership skills. During the semester, the students work on these skills through the activity titled “discussion leader.” This activity is represented by the weekly team-based presentations developed and led by the students. At the start of the semester, students are divided into groups within which the team members collaborate on preparing a presentation related to a case study or an article assigned for that week. Each week, one team leads a presentation and the class discussion based on the assignment centered on one of the EPHS discussed that week. Again, health equity becomes one of the main themes emphasized during these discussions. Leading the discussion allows the students to collaborate on a common task as a team, learn from each other, and advocate for disadvantaged populations. These activities also target the “Professionalism, networking, and personal work ethic” component of the model by letting the students practice their professional communication and presentation skills.

### Critical thinking skills

Working on the class assignments, participating in class activities, and leading the discussions with the focus on the ten EPHS and health equity, evidence-based practice, analysis of various public health problems, their causes and solutions, and creating new solutions to existing problems allows for a gradual mastery of critical thinking and problem-solving skills over the course of the semester. Analysis of various real-life problems allows the students to distinguish the role of behavioral, social, and environmental factors on the health status of selected populations. Weekly, the instructor provides written feedback on the extent to which students sufficiently addressed the questions related to the aspects mentioned above, which helps them to gradually build the skills necessary to work on the final class project.

All the components of the model discussed above, when combined and incorporated in the classroom, allow the demonstration of knowledge from various public health-related disciplines and the integration of this knowledge. Specifically, case studies, article reviews and class activities and discussions demonstrate the relatedness of the concepts from various public health domains. Examination of a central issue, problem, or topic allows the students to strengthen their ability to connect the concepts from more than one discipline and practice synthesis skills (12). Such an approach reflects the interdisciplinary nature of the BSPH program that focuses on students' exposure to a less fragmented and more cohesive educational process (12). Moreover, it promotes mastery of knowledge related to public health domains and cross-cutting areas and understanding of the relationship between public health theory and practice.

### Public health problem and solution paper and presentation

The final assignment, titled Public Health Problem and Solution Paper is the culmination of students' learning experiences in the BSPH program. This assignment being a part of the capstone course stands separately from all the other assignments as, according to the American Association of Colleges and Universities standards, it represents an example of high-impact educational practices (13). In this capstone course, this assignment consists of the following parts: each student selects a public health problem, describes the scope of the problem, identifies the population affected by this problem, analyzes causes of this problem, identifies stakeholders relevant to that problem and proposed solution, and proposes a public health solution to address this problem with the explanation of how this solution would be incorporated within the public health and/or healthcare delivery system. Moreover, in the Public Health Problem and Solution paper, each student needs to emphasize how this particular solution would address health disparity affecting the population that they identified and promote health equity.

The students start working on the final project early in the semester by selecting a topic that interests them. The selection of a topic is driven by the availability of public health surveillance system created to gather the data on this particular topic. The data generated by the surveillance system and which the student is planning to use has to be recent and provide information regarding the distribution of this problem in a specific population. This requirement results in a more thorough approach by the student when selecting the topic. Even if the topic interests the students, the availability of a public health surveillance system that exists to assess the problem is the limiting factor that requires further research. As a result, the students might change their initial topic to another in order to address this requirement.

Once the instructor approves the topic, the students move to the next stage of this project- creating an outline of the final paper in which the students have to provide the main details of each part of the paper listed above. During this stage, the instructor assesses the students on how well each component of each part of the paper relates to the overall topic and how well these components are aligned with one another. Students might need additional guidance regarding the proposal of the solution that addresses the selected problem. This is quite understandable considering the fact that creating a solution corresponds to the very last category of the cognitive process dimension of the revised Bloom's taxonomy, which tends to be more cognitively complex compared to lower levels (14). At the same time, the students are expected to synthesize previously learned knowledge into a new product (14). For this reason, constructive feedback from the instructors and some suggestions regarding the feasibility of a proposed solution and its link to the preceding parts is necessary for the students to navigate this part of the final paper.

Another part that could be somewhat challenging for the students to describe is how the solution that they proposed would be incorporated within the public health and/or healthcare delivery system, given the “complexity of designing and delivering public health services.” (15) Again, weekly assignments, class activities and discussion and the instructors' emphasis on details of utilization of public health and healthcare delivery system to implement various solutions provided in the description of the case studies and articles help the students to notice these important details and use similar techniques when describing the implementation of their solutions. Another useful tool that helps the students to think through the details of the implementation of their solutions is creating diagrams and chronological timelines of the situations described in the case studies and articles. Such techniques guide students in identifying the elements of the delivery system necessary to integrate the solutions proposed in the case studies and articles and visualize the links among these elements.

In the final class product, the students have to demonstrate the ability to select and describe a problem using existing data, identify the population affected by this problem, analyze the causes of the problem supporting their claims with scientific literature, describe the role of each of the identified stakeholder related to the problem and its solution, create a public health solution based on evaluation of the existing evidence of a previously created functional solution, and evaluate the created solution in relation to how it addresses health disparity and health equity concept. Using a detailed rubric, students are assessed on how well each of these parts is addressed in the paper and how well these parts are linked together into one cohesive paper.

At the end of the semester, the students also create PowerPoint presentations containing the main points of their papers and deliver an oral presentation to their peers. This assignment further masters the skill of professional communication by presenting the information to a larger audience. The students are then assessed based on the quality of the presentation of the content of the final paper and their public speaking skills.

As described in Figure 1, the Public Health Problem and Solution assignment aligns with the model utilized in this course. The students master the skills to interpret quantitative data and utilize scientific evidence to develop a public health-related solution, further explore EPHS, specifically EPHS #1 and #5 and explain how evidence is utilized across the EPHS, and further explore the concept of health equity. By working on this paper, students continue mastering critical thinking and problem-solving skills and move beyond basic levels of the revised Bloom's Taxonomy to the highest, most challenging level. Finally, aligned with the main model in this class, the students demonstrate their ability to synthesize and integrate knowledge acquired from the core public health courses.

## Discussion

Being a course in which the “students have opportunities to integrate, synthesize, and apply knowledge through cumulative and experiential activities,” (4) the capstone experience offers a unique way to strengthen the knowledge and skills acquired by the students throughout the program. With different capstone courses described in the literature (16–18), the Modern Public Health Problems and Solutions course in the BSPH program at the University at Buffalo specifically prepares the students to target health disparities by mastering the students' ability to rely on evidence-based practices mastering the cross-cutting skills identified by CEPH, therefore, contributing to the knowledge base for the design of future courses of similar nature. The course capitalizes on achieving health equity through addressing health disparities, an essential skill for public health professionals. Together with the class activities, case studies, article reviews, and the Public Health Problem and Solution paper and presentation help prepare the students to address “real world” issues when they become public health professionals.

We also recognize some challenges that exist in preparing the students for the “real world” challenges. First, some students had difficultly and needed additional guidance with linking the parts of the final paper together in one logical framework. We are planning on addressing this issue by including the tool that would encourage systems thinking approach (4). Based on the CEPH criteria, such tools could include a concept map that would visualize the complicated connections between the causes of a public health problem, stakeholders, parts of the public health or health care-related delivery system, and the health equity concept.

To make the class environment more supportive, similarly to the capstone course at the University at Albany (18), we offered office hours as an additional support option for students who had some difficulty while working on the class assignments. In addition, to promote equity within the classroom, we also utilized the Transparency in Learning and Teaching (TILT) templates (19) to organize the description of the assignments in this class. Transparent instructions with a formulated goal of the assignment and its relevance for students' classroom experience have been shown to be highly beneficial for students' success, particularly for underrepresented students and students with a disadvantaged background (20). Knowing the goal of each assignment and its relevance increased the engagement of students with the class material and made each assignment more focused. We are planning to continue using these templates in this course as its utilization, together with the class model, helped the students gradually develop and master the skills of integration and synthesis of previously acquired knowledge.

## Conclusion

To accomplish the fundamental task of achieving health equity, public health programs should focus their training of the next generation of public health professionals on recognizing the presence or lack of health equity and developing and mastering the ability to participate in the efforts to address the needs of disadvantaged populations. To be able to face challenges when pursuing this important goal, that the graduates of public health programs should be knowledgeable not only about the discipline-related concepts but also be able to interpret scientific evidence, utilize evidence-based strategies to address public health problems, have strong teamwork, leadership, professional communication, critical thinking, and problem-solving skills. We are optimistic that our experience of framing the capstone course will serve as an example for similar courses in other undergraduate public health programs. Centering the coursework on mastering these skills as well as prioritizing the role of health equity in public health activities will prepare a competent and skilled workforce ready to lead the national public health goal of improved and equitable population health.

## Data availability statement

The original contributions presented in the study are included in the article/supplementary files, further inquiries can be directed to the corresponding author/s.

## Ethics statement

Ethical review and approval was not required for the study on human participants in accordance with the local legislation and institutional requirements. Written informed consent for participation was not required for this study in accordance with the national legislation and the institutional requirements.

## Author contributions

AM and KA were the instructors for the Modern Public Health Problems and Solutions course. SP is the Assistant Dean, Director for Undergraduate Public Health Programs who oversees the Capstone course. All authors directly contributed to the development, writing, and review of the manuscript.

## Conflict of interest

The authors declare that the research was conducted in the absence of any commercial or financial relationships that could be construed as a potential conflict of interest.

## Correction note

A correction has been made to this article. Details can be found at: 10.3389/fpubh.2025.1750920.

## Publisher's note

All claims expressed in this article are solely those of the authors and do not necessarily represent those of their affiliated organizations, or those of the publisher, the editors and the reviewers. Any product that may be evaluated in this article, or claim that may be made by its manufacturer, is not guaranteed or endorsed by the publisher.

## References

[1] USDHHS. Health Equity in Healthy People 2030. Available online at: https://health.gov/healthypeople/priority-areas/health-equity-healthy-people-2030 (accessed May 11, 2022).

[2] CDC. 10 Essential Public Health Services 2020. Available online at: https://www.cdc.gov/publichealthgateway/publichealthservices/essentialhealthservices.html (accessed on March 18, 2021).

[3] CDC. Health Equity 2022. Available online at: https://www.cdc.gov/chronicdisease/healthequity/index.htm (accessed June 6, 2022).

[4] CEPH. Accreditation Criteria: Schools of Public Health & Public Health Programs 2021. Available online at: https://media.ceph.org/documents/2021.Criteria.pdf (accessed June 11, 2022).

[5] Kiviniemi MT Przybyla SM. Integrative approaches to the undergraduate public health major curriculum: strengths, challenges, and examples. Front Public Health. (2019) 7:106. 10.3389/fpubh.2019.00106 31114779 PMC6503149

[6] SPHHP. Requirements and Curriculum. Available online at: https://publichealth.buffalo.edu/home/education/undergraduate-and-minors/public-health/requirements-and-curriculum.html (accessed June 9, 2022).

[7] de Beaumont Foundation and Association of State and Territorial Health Officials (ASTHO). Public Health Workforce Interests and Needs Survey: 2017 Findings. Available online at: https://phwins.org/most-recent-findings/ (accessed June 20, 2022).

[8] Turnock BJ. Public Health: What it is and How it Works. Sixth edition. Burlington, Massachusetts: Jones & Bartlett Learning (2016).

[9] Andreasen RJ. Integrating experiential learning into college of agriculture capstone courses: implications and applications for practitioners. NACTA J. (2004) 48:52–7.

[10] Brownson RC Fielding JE Maylahn CM. Evidence-based public health : a fundamental concept for public health practice. Annu Rev Public Health. (2009) 30:175–201. 10.1146/annurev.publhealth.031308.100134 19296775

[11] Hunting K Gleason BL. Essential Case Studies in Public Health: Putting Public Health into Practice. 1 ed. Burlington, MA: Sudbury Jones & Bartlett Learning, LLC (2011).

[12] Jacobs HH. Interdisciplinary Curriculum: Design and Implementation. Alexandria, Virginia: ASCD, Association for Supervision and Curriculum Development (1989).

[13] AAC&U. High-Impact Practices 2022. Available online at: https://www.aacu.org/trending-topics/high-impact (accessed June 13, 2022).

[14] Anderson KDR. A Taxonomy for Learning, Teaching, and Assessing : A Revision of Bloom's Taxonomy of Educational Objectives. Pearson; 1st edition (December 19, 2000) (2001), 336.23664405

[15] Liburd LC Hall JE Mpofu JJ Williams SM Bouye K Penman-Aguilar A. Addressing health equity in public health practice: frameworks, promising strategies, and measurement considerations. Annu Rev Public Health. (2020) 41:417–32. 10.1146/annurev-publhealth-040119-094119 31900101

[16] Chorazy ML Klinedinst KS. Learn by doing: a model for incorporating high-impact experiential learning into an undergraduate public health curriculum. Front Public Health. (2019) 7:31. 10.3389/fpubh.2019.00031 30863743 PMC6399422

[17] Harver A Zuber PD Bastian H. The capstone eportfolio in an undergraduate public health program: accreditation, assessment and audience. Front Public Health. (2019) 7:125. 10.3389/fpubh.2019.00125 31214557 PMC6554422

[18] Fitzpatrick VE Mayer C Sherman BR. Undergraduate public health capstone course: teaching evidence-based public health. Front Public Health. (2016) 4:70. 10.3389/fpubh.2016.00070 27148516 PMC4834356

[19] TILT HE. TILT Higher Ed Examples and Resources. Available online at: https://tilthighered.com/tiltexamplesandresources (accessed June 13, 2022).

[20] UNLV. Recent Findings: Transparency in Learning and Teaching in Higher Education 2015. Available online at: https://www.unlv.edu/sites/default/files/page_files/27/Provost-TILT-ResearchSummary.pdf~

