# Correction: Modern public health problems and solutions: an undergraduate capstone course to prepare the next generation of public health practitioners to enhance health equity

**DOI:** 10.3389/fpubh.2025.1750920

**Published:** 2025-12-01

**Authors:** Frontiers Production Office

**Affiliations:** Frontiers Media SA, Lausanne, Switzerland

**Keywords:** capstone course, undergraduate public health education, bachelor of science public health, health equity, public health workforce

The title of this article was erroneously given as: “Modern public pealth problems and solutions: An undergraduate capstone course to prepare the next generation of public health practitioners to enhance health equity”. The correct title of the article is “Modern public health problems and solutions: An undergraduate capstone course to prepare the next generation of public health practitioners to enhance health equity”.

The original version of this article has been updated.

